# Dietetic Nuggets

**Published:** 1904-10

**Authors:** 


### DIETETIC NUGGETS.

"Generous feeding" ought not to, but generally does, mean gormandizing.

There is often more depending on how one eats than upon what he eats.

The so-called "hearty meal" is more frequently the cause assigned in cases of sudden death than it ought to be.

"Acute indigestion" is the fashionable, or at least polite, name for over-feeding—for which the unpolished English is *gluttony*.

The punishment of dietetic sins is not always swift, but it is remarkably certain to put in its appearance.

Pepsin is a cowardly prop.

So live that when the summons comes to join the hungry caravan that moves to the inviting spread in the dining-room you may be quite competent to secrete your own pepsin.

Time is the essence of all contracts; every meal is a new contract with one's stomach; therefore be on time. In other words *eat regularly.* There are many important dietetic rules; none more important than this. The man who eats one meal to-day and four to-morrow, or who dines at any convenient hour, all the way from 5 p. m. to midnight, is on the direct road to digestive purgatory.

The fashionable black coffee after dinner hasn't a redeeming feature in its favor. It is neither food nor drink. It is merely a popular form of taking a two-grain dose of a nervo-narcotic—*caffein.* If taken for its several effects on the nervous system it is neither more or less than a tipple. As between the two a like sip of any good wine would be less harmful. The best stomachs, livers and nerves in the world can not very long withstand such constant nagging.

If you "have no appetite," no unmistabable desire for a meal when ready, *Keep away from the table.* To do otherwise is to choke with more fuel a fire that has gone out.

A piece of pie is not necessarily a death-warrant. It depends on what it is made of and how made. Pie proper should represent an unobjectionable combination of fruit and bread.

The word "pastry," however, covers a multitude of dietetic sins. Flour and fat rolled into a couple of soggy layers, between which spiced meats chopped with more fat, mingled with raisins and other fruits, and moistened with cognacs—this may be *pastry,* but it is a libel on *pie.*

A crust made reasonably tender with sweet cream, olive oil, or fresh butter, or with half butter and half beef suet (the soft variety)

with sufficient baking powder or cream of tartar and soda to make it *light and porous,* filled with good, wholesome fruit or berries—this is *pie ;* and it is quite as digestible and harmless as the ordinary's baker's loaf.

It is time the pie libel was relegated to the limbo of other lies.—*Dietetic and Hygienic Gazette.*

